# Immunomodulatory Drugs in the Treatment of Hidradenitis Suppurativa—Possibilities and Limitations

**DOI:** 10.3390/ijms23179716

**Published:** 2022-08-26

**Authors:** Zuzanna Świerczewska, Miłosz Lewandowski, Agnieszka Surowiecka, Wioletta Barańska-Rybak

**Affiliations:** 1 Department of Dermatology, Venereology and Allergology, Faculty of Medicine, Medical University of Gdansk, Smoluchowskiego 17, 80-214 Gdansk, Poland; 2 Faculty of Medicine, Medical University of Gdansk, Marii Skłodowskiej-Curie 3a, 80-210 Gdansk, Poland; 3 East Center of Burns Treatment and Reconstructive Surgery, District Hospital in Łęczna, ul. Krasnystawska 52, 21-010 Łęczna, Poland

**Keywords:** skin diseases, hidradenitis suppurativa, acne inversa, biological therapies, target therapies

## Abstract

Hidradenitis suppurativa, also known as acne inversa, is a chronic, progressive, debilitating, recurrent inflammatory skin disease characterized by the occurrence of very severe, persistent, painful nodules, abscesses, and fistulas, most commonly found in the skin folds of the axilla, groin, gluteal, and perianal areas. Treatment is rather difficult and typically requires the use of multiple modalities. Regardless of the presence of several therapeutic options, treatment often turns out to be ineffective or poorly selected concerning the clinical picture of the disease. Thus, the search for new biologics and other target treatments of hidradenitis suppurativa is ongoing. The safety and efficacy of adalimumab, still the only U.S. Food and Drug Administration approved biologic in the hidradenitis suppurativa treatment, paved the way for new drugs to be compared with it. Several more drugs with new immunological targets are currently under investigation for the treatment of acne inversa. The aim of the article was to present the current and future targets of acne inversa treatment, simultaneously providing insights into the molecular pathomechanisms of the disease.

## 1. Introduction

Hidradenitis suppurativa (HS), also known as acne inversa, is a chronic, progressive, debilitating, recurrent inflammatory skin disease characterized by the occurrence of very severe, persistent, painful nodules, abscesses, and fistulas, most commonly found in the skin folds of the axilla, groin, gluteal, and perianal areas [1]. Several studies tried to assess the general prevalence of hidradenitis suppurativa; nonetheless, due to the demographic diversity of the surveyed populations and different research methodologies, the results of the studies differ significantly, and presently, the prevalence range is estimated at 0.00033–4.1% [2]. Nevertheless, it is worth noticing that the prevalence of the disease in the majority of the research does not exceed 1% and a result as high as 4% occurred only in a single prospective study conducted in 1992 in Denmark on 507 people [3]. The highest incidence of the disease is noted in the 20–40 age range group, whereas the prevalence declines in those over 50 years of age. Even though it is a rare phenomenon, and the first symptoms of the disease occur most often in the twenties, the disease can also affect pediatric patients, which might be related to the family history of the disease. The most prevailing comorbidities in patients with HS are cardiovascular disease, spondylarthritis, subclinical atherosclerosis, inflammatory bowel disease, sexual dysfunction, and mental disorders. Treatment is rather difficult and often requires the use of multiple therapeutic options. The choice of an appropriate treatment for HS should depend on the clinical severity of the disease. There are numerous therapeutic options for patients with acne inversa, including a pharmacological and surgical approach. They should be supported by adjuvant therapy based on lifestyle modifications such as weight loss or smoking cessation, which are known to exacerbate the course of the disease [1,4]. For mild stages of HS, topical therapy with antibiotics, keratolytic, or intralesional corticosteroids is advised. Patients with moderate to severe lesions are typically offered systemic antibiotics for 3 months or biologic therapy with the only approved drug in HS, adalimumab. Retinoids, systemic corticosteroids, metformin, and cyclosporin A have also found their use in the treatment of acne inversa. Chronic, severe lesions, and unresponsive to previous pharmacological therapy are recommended to be treated with surgery [1,5,6]. Regardless of the presence of several therapeutic options, treatment often turns out to be ineffective or poorly selected concerning the clinical picture of the disease. Thus, the search for new biologics and other target treatments of HS is intensively ongoing.

The aim of this article was to present the current and future targets of HS treatment, simultaneously providing insights into the molecular pathomechanisms of the disease.

## 2. Pathogenesis

The pathogenesis of hidradenitis suppurativa is multifactorial and has not been fully established yet. Factors such as genetics, lifestyle, hormonal status, environment, and microbiota all seem to play a role in the process. They cause immune activation that leads to chronic inflammation. In hidradenitis suppurativa, intertriginous regions around the pilosebaceous-apocrine unit are where the skin lesions develop [1]. Mechanical friction within the skin fold causes local cell damage, which results in the production of cellular damage-associated molecular patterns (DAMPs) and entering microbiological components into the skin. These processes lead to the activation of local resident immune cells, mainly macrophages, which secrete pro-inflammatory cytokines such as IL-1β and TNF as a result of pattern recognition receptors (PRR) (such as toll-like receptors (TLR) or NOD-like receptors (NLR)) stimulation by DAMPs [7,8]. Moreover, the role of the inflammasome, which is required for the detection of DAMP and the subsequent post-translational activation of IL-1β and IL-18, is already present at this stage. Activation of the pathway of the currently best known inflammasome—NLRP3 (NOD-like receptor family, pyrin domain-containing 3) is enhanced in skin lesions of patients suffering from HS, leading to the enhanced expression of several inflammatory mediators such as interleukin (IL)-1β, IL-17, caspase-1, S100A8, and S100A9 [9,10]. According to research, IL-1β and TNF are pleiotropic cytokines that influence various cell types and have in some measure overlapping outcomes [11,12]. The production of chemokines by fibroblasts, such as CXCL1 and CXCL6 being the most leading, is induced by secreted IL-1β. On the other hand, TNF induces chemokines production such as CXCL8, CXCL11, CCL20, and CCL2 in keratinocytes. Consequently, the chemokine release and endothelial cell activation result in a significant immune cell infiltration of the forming lesions [13]. The most prevalent cells in HS lesions are neutrophilic granulocytes, macrophages, dendritic cells, and T lymphocytes [14,15]. T17 cells and T1 cells, supported by the dendritic cell mediators IL-23 and IL-12, produce their particular cytokines IL-17 and interferon γ (IFNγ), respectively. IFNγ, the main cytokine of TH1 cells, stimulates dermal endothelial cells in the HS lesion, allowing immune cells from the circulation to infiltrate and stimulate macrophages and tissue cells, and also the production of TH1-attractive chemokines such as CXCL10, resulting in a positive feedback loop [16,17]. IL-17 increases the synthesis of certain chemokines (such as CCL20, CXCL1, and CXCL8), cytokines (such as G-CSF and IL-19 (which enhances the effects of IL-17)), and epidermal antimicrobial proteins (AMPs) [18,19]. Although IL-17 produces relatively mild cell responses on its own, it works synergistically with TNF, IL-22, and IFN [1]. The consequence of the action of interleukins, chemokines, hyperplasia, and hyperkeratosis of the infundibular epithelium of the hair, causing follicular occlusion, is follicular rupture, inflammatory nodule fistulas, and abscesses formation [1]. Interestingly, skin in acne inversa also has the ability to express the anti-inflammatory mediator, IL-10. It is induced by the autocrine action of pro-inflammatory cytokines, including TNF in macrophages. By suppressing the production of pro-inflammatory cytokine by monocytes and macrophages, IL-10 decreases immune responses and thus limits the activation of T cells [9,20]. Although IL-36 is not under the highest interest of scientists working on HS, it has been proved that IL-36α, -β, and -γ concentrations are considerably greater in HS patients’ lesions compared to healthy subjects. Moreover, S. Hessam et al. showed that IL-36 receptor antagonist (IL-36RA) was significantly dysregulated in lesional and perilesional hidradenitis suppurativa (HS) skin, so IL-36 signaling may be unrestricted in these patients [21].

## 3. Treatment

The treatment of HS is based on multiple options including topical, systemic, surgical, and combined therapies. Despite the presence of several therapeutic modalities, treatment often turns out to be ineffective, hence the search for novel alternatives is ongoing.

### 3.1. TNF-α Inhibitors

#### 3.1.1. Adalimumab

Adalimumab (ADA) is the only registered biologic in the treatment of moderate to severe hidradenitis suppurativa. It is a fully human recombinant IgG1 monoclonal antibody against TNF-α [6]. ADA is administered as a 160-mg loading dose injected subcutaneously at week 0, followed by an 80-mg subcutaneous injection at week 2, and 40-mg subcutaneous injections weekly thereafter. It binds soluble TNF-α, preventing it from interacting with TNFR1- and TNFR2 cell receptors. Furthermore, it alters the amounts of adhesion molecules responsible for leukocyte migration and decreases serum concentrations of CRP (C—reactive protein), the erythrocyte sedimentation rate, and IL-6. It also decreases the serum levels of metalloproteinases by 50% (MMP-1 and MMP-3) [22]. Several studies proved its safety and efficacy for the treatment of moderate to severe acne inversa [23,24,25]. According to research, ADA has the ability to decrease the production of cytokines in the lesional skin of patients with HS, specifically IL-1β and CXCL9 [26].

To this day, the most significant phase III trials for the evaluation of the efficacy and safety of ADA in acne inversa treatment are the PIONEER I and PIONERR II [24]. The study included a total of 633 patients (*n* = 307 and *n* = 326, respectively, in PIONEER I and PIONEER II) who were given either a placebo or loading doses of adalimumab for the first 2 weeks, and a lower dose weekly thereafter. The design for two trials was nearly indistinguishable. The main distinction was the concomitant oral tetracycline class antibiotics allowed in PIONEER II. The primary efficacy end point was HiSCR (hidradenitis suppurativa clinical response), defined as more than a 50% reduction in the number of inflammatory lesions (the total count of abscesses and inflammatory nodules), no increase in the number of abscesses, and no increase in the number of draining fistulas. For patients treated with adalimumab, the HiSCR achievement rate was significantly higher compared with those treated with a placebo (41.8 vs. 26% in PIONEER I and 58.9 vs. 27.6% in PIONEER II). Moreover, in the PIONEER II, ADA was demonstrated to be remarkably more effective than the placebo in secondary outcomes, which included disease severity, pain reduction, and the number of skin lesions. What is more, a statistically significant improvement in quality of life (DLQI) was observed in this group. The percentage of patients reporting minor side effects or severe adverse events were comparable across the placebo-treated and adalimumab-treated groups in both trials.

In addition, Bechara et al. investigated the safety and efficacy of adalimumab in conjunction with surgery in moderate to severe HS in a phase IV trial [27]. In this study, a total of 206 patients were randomized 1:1 to receive 40mg of adalimumab or placebo during presurgery (12 weeks), perioperative (2 weeks), and postoperative (10 weeks) periods. The proportion of patients achieving a clinical response across all body regions at week 12 was the primary end point. Significantly more patients receiving adalimumab (49 of 103) vs. placebo (35 of 103) achieved a clinical response across all body regions at week 12. Adverse reactions were reported in 72% in the ADA group and 67% in the placebo group. Noteworthy, no increased risk of postoperative wound infection or complication was observed. Currently there are two phase IV trials investigating the safety and efficacy of ADA [28,29].

#### 3.1.2. Infliximab

Infliximab (IFX) is a chimeric mouse/human monoclonal IgG1 antibody against TNF-α. It prevents TNF-α from attaching to its receptors by binding to the soluble and transmembrane forms of it [30]. According to the European S1 guidelines [6], IFX is administered on week 0, 2, 6, and then regularly every 8 weeks. It is recommended as a 2nd line therapy in moderate to severe HS unresponsive to adalimumab.

In a phase II double-blind trial by Grant et al., patients received infliximab, or a placebo for 8 weeks followed by an open-label crossover treatment phase and an observational phase. The primary endpoint was based on the HS severity index (HSSI) and the secondary endpoints included dermatology life quality index (DLQI), visual analog scale (VAS), and physician global assessment (PGA) scores [31]. Patients treated with IFX responded with a 50% or greater decrease in HSSI compared to the placebo. After 8 weeks of treatment, significant improvements in the infliximab group compared to the placebo group in terms of the mean DLQI change and mean PGA scores were also observed. In the placebo group, patients treated with infliximab after crossover responded similarly to the original infliximab group. Most adverse events were mild and expected.

Other studies also proved the efficacy and safety of IFX in HS [32,33]; however, it is not under investigation in any ongoing clinical trials.

#### 3.1.3. Etanercept

Etanercept (ETN) is a fusion protein that consists of two identical chains of the recombinant human TNF-receptor p75 monomer combined with the Fc domain of human IgG1. It has the ability to bind and inactivate TNF [34]. Thus far, etanercept has been approved by the FDA for use in several conditions including rheumatoid arthritis (RA), ankylosing spondylitis, juvenile idiopathic arthritis, psoriatic arthritis, and plaque psoriasis [35]. Nonetheless, studies investigating the use of ETN in HS reveal diverse results. In a prospective open-label phase II research by Giamarellos-Bourboulis et al., 10 patients were injected with 50 mg of etanercept subcutaneously once a week for 12 consecutive weeks [36]. The VAS significantly decreased in seven patients at week 12 and in six patients at week 24. A decrease in local pain at the site of lesions after week 4 was observed in each patient. Overall, the therapy was well tolerated, and the results were notable.

Opposite results have been achieved in research by Lee et al., which failed to demonstrate the efficacy of 50 mg of etanercept administered subcutaneously weekly for 12 weeks [37]. Out of 15 patients included in the study, only 3 achieved 50% or greater improvement in PGA at week 12. A slight improvement was observed in DLQI (from a median of 19 to 15; *p* = 0.02) and pain scores (from 6.4 to 4.1; *p* = 0.08), however, not more than 29% of patients reported moderate improvement in their disease.

What is more, there has been only one randomized, double-blind, placebo-controlled trial that also did not prove the efficacy of the treatment with ETN in acne inversa [38]. Twenty patients with moderate to severe HS enrolled in the study were administered 50 mg of ETN or placebo twice a week for the following 12 weeks. After 12 weeks, each patient received 50 mg of open label ETN subcutaneously twice a week for another 12 weeks. The primary endpoint was defined as a physician global assessment (PGA) of clear or mild at week 12. Secondary endpoints included the patient global assessment of lesions, patient-reported pain, and DLQI. No statistically significant differences in any of the above-mentioned endpoints were obtained at week 12 and 24. All things considered, the evidence supporting the use of etanercept in acne inversa is lacking; nevertheless, adequate doses may have not been utilized in order to obtain satisfactory effects.

#### 3.1.4. Golimumab

Golimumab is a human anti-TNFα monoclonal antibody (IgG1κ). Heavy and light variable chain regions (Fab) of GOL are made of an amino acid sequence that is very similar to that of the human one. Its Fab region may bind to both sTNF and tmTNF since it is bivalent and specific for human TNF. As a result, it reduces TNF binding to receptors and its circulation [39]. Golimumab has been so far approved by U.S. Food and Drug Administration for the treatment of adult patients with moderately to severely active rheumatoid arthritis (RA), in combination with methotrexate, active psoriatic arthritis (PsA) in adults, alone or in combination with methotrexate, and active ankylosing spondylitis in adults (AS) [40]. Antonio Tursi in a letter to editor described a case report of a patient who suffered from hidradenitis suppurativa Hurley stage II, inflammatory bowel disease grade 2 (according to endoscopic Mayo score), and pyostomatitis vegetans. The patient started anti-TNF-α therapy with golimumab (200 mg subcutaneously followed by 100 mg s.c. every 4 weeks), together with amoxicillin-clavulanate 2 g/day for 2 weeks: dermatological, as a result of treatment, oral lesions disappeared within two months of therapy, and colonoscopy complete mucosal healing. During the continued treatment with golimumab, oral and dermatological lesions did not recur, and UC was in clinical remission [41]. In another case report described by H.H. van der Zee et al., golimumab (50 mg subcutaneously once a month for 8 months) showed no effectiveness in improving skin lesions in a patient with severe HS and comorbid psoriatic arthritis, however, showed great improvement selectively in arthritis treatment [42]. A comparison of the two mentioned may suggest that a higher dosing of golimumab may be needed for HS treatment.

#### 3.1.5. Certolizumab

A humanized antibody (IgG4) called certolizumab pegol is a polyethylene glycolylated (PEGylated) fragment antigen binding (Fab) that interacts with both soluble and trans-membrane TNF. As a result, the TNF-dependent adhesion of molecules, chemokines, and pro-inflammatory mediators becomes downregulated [43]. Certolizumab has been so far approved by the U.S. Food and Drug Administration for the only one dermatological condition with moderate to severe plaque psoriasis for adults being candidates for systemic therapy or phototherapy [44]. It offers an alternative to other anti-TNF-inhibitor biologics in pregnant patients, due to its inability to cross the placenta [45]. There are currently four studies describing a total of five patients (three women including two pregnant, and two men) who have been shown to be effective in treating HS with certolizumab (200 mg or 400 mg; once a week or once every 2 weeks) [46,47,48,49]. However, in one other retrospective study, authors described two cases of patients suffering from HS with no response to a certolizumab 200 mg twice monthly treatment [50]. The effectiveness of certolizumab pegol in hidradenitis suppurativa treatment across the published studies is promising nonetheless, but still insufficient, and therefore the role of certolizumab pegol in HS warrants further investigation.

### 3.2. IL-1 Inhibitors

#### 3.2.1. Anakinra

Anakinra (ANR) is a recombinant IL-1 receptor antagonist that competitively inhibits the binding of IL-1α and IL-1β to the IL-1 type 1 receptor. It is FDA-approved for the treatment of rheumatoid arthritis (RA) and neonatal-onset multisystem inflammatory disease [51]. It has been studied in a double-blind, randomized, placebo-controlled clinical trial with a 12-week treatment phase and a 12-week follow-up phase [52]. Twenty patients enrolled in the study were randomized to receive subcutaneous injections of 100 mg ANR or placebo once daily for 12 weeks. The primary endpoint was the effect of ANR on HS severity and the secondary endpoints were the period of time to a new exacerbation and the production of cytokines. After 12 weeks, the disease activity score was reduced in 20% of the placebo group in comparison to 78% of the study group. As far as the cytokines’ levels, the production of interferon-γ in the study group was decreased, and the production of interleukin 22 was increased. Furthermore, a notably longer time until the new exacerbation was observed after the ANR therapy. It is also worth mentioning that no serious adverse events were noticed. Therefore, the authors stated that injections with 100 mg anakinra might be a safe and effective treatment option for patients with acne inversa.

#### 3.2.2. Bermekimab

Bermekimab is a recombinant human immunoglobulin G1 kappa (IgG1k) monoclonal antibody that binds with high affinity and selectivity for human IL-1α. Hence, it is an effective blocker of IL-1α activity. Thus far, its safety and efficacy have been investigated in two clinical trials [53,54]. In 2018, Kanni et al. presented the results of a randomized study on patients with moderate to severe HS not eligible for adalimumab [54]. Twenty patients were randomized to receive either a placebo or bermekimab for 12 weeks. The drug was administered every 2 weeks at a dose of 7.5 mg/kg. The primary endpoint was the hidradenitis suppurativa clinical response (HiSCR) score at the end of the treatment, which was met in 10% of placebo and 60% of bermekimab patients (*p* = 0.035). The authors reported no serious adverse events after bermekimab.

Two years later, in 2020, Gottlieb et al. conducted a phase II open-label study in patients with moderate to severe acne inversa who have failed (group A) or were naive (group B) to prior anti-TNF treatment [53]. Bermekimab was administered subcutaneously at a dose of 400 mg once a week. After 12 weeks, HiSCR was reached in 63% of patients in group A and 61% of patients in group B. What is more, a statistically significant reduction in the visual analogue scale (VAS) pain score was observed by 54% (*p* < 0.001) and 64% (*p* < 0.001) in group A and B, respectively.

Currently, bermekimab is under investigation in one randomized, placebo-controlled, double-blind phase II clinical trial [55].

#### 3.2.3. Canakinumab

Canakinumab is a human monoclonal antibody against IL-1β. In hidradenitis suppurativa, it has been given as a 150 mg subcutaneous weekly dose. Up to this point, no large clinical trials investigated its safety and efficacy in HS and the only data come from single case reports or case series [56,57,58]. The results of the treatment with canakinumab are relatively varied, hence further research is needed to prove its validity in HS. A summary of significant clinical studies concerning the IL-1 inhibitors in HS are presented in Table 1.

### 3.3. IL-17 Inhibitors

#### 3.3.1. Secukinumab

Secukinumab is a human monoclonal immunoglobulin G1 kappa (IgG1k) antibody that selectively binds and neutralizes IL-17. It has been approved by the FDA in the treatment of psoriatic arthritis, plaque psoriasis, and ankylosing spondylitis [59]. Two open-label clinical trials investigated the use of secukinumab in acne inversa showing promising results; however, both included a small number of subjects and indicated the need for further studies [60,61]. The authors of both pieces of research used weekly injections of secukinumab 300 mg subcutaneously.

Currently, there are three ongoing phase III trials with much larger study groups that will allow for a better assessment of the drug’s effectiveness in HS [62,63,64].

#### 3.3.2. Bimekizumab

Bimekizumab is a humanized IgG monoclonal antibody that selectively inhibits IL-17A and IL-17F. In 2021, a double-blind, placebo-controlled, phase 2 randomized clinical trial that included 90 patients with hidradenitis suppurativa demonstrated very encouraging results. Patients were randomized 2:1:1 to receive bimekizumab (640 mg at week 0, then 320 mg every 2 weeks), placebo, or adalimumab (160 mg at week 0, 80 mg at week 2, and 40 mg every week for weeks from 4 to 10). Bimekizumab achieved a higher HiSCR (hidradenitis suppurativa clinical response) rate (57.3% in the bimekizumab group and 26.1% in the placebo group) and demonstrated greater clinical improvements compared with the placebo at week 12. Serious adverse reactions were reported in 2 of 46 bimekizumab-treated subjects, 2 of 21 placebo-treated, and 1 of 21 adalimumab-treated [65]. According to a meta-analysis by CH Huang et al., among all immunomodulatory drugs, bimekizumab has been found to be effective in improving both the severity of the disease and the quality of life [66]. At the time of writing, a few phase III clinical trials are underway to investigate the potential of bimekizumab in HS [67,68].

#### 3.3.3. Brodalumab

Brodalumab is a fully human monoclonal antibody that binds to the IL-17 receptor A and thus inhibits the biological effect of IL-17A, IL-17C, and IL-17F isoforms. Approved by the FDA for the treatment of plaque psoriasis, several researchers also tried to investigate its validity in acne inversa. In 2020, Frew et al. published the results of their cohort study research in which the authors tried to assess the safety, tolerability, and clinical response of brodalumab at weeks 12 and 24 in moderate to severe HS [69]. Ten patients were administered brodalumab 210 mg/1.5 mL subcutaneously at weeks 0, 1, and 2, and every 2 weeks thereafter until week 24. Hidradenitis suppurativa clinical response (HiSCR), Sartorius, international hidradenitis suppurativa severity scoring system (IHS4), ultrasonography, and skin biopsies were assessed. All patients achieved HiSCR and at week 12, 80% achieved an IHS4 category change. An improvement in pain, itch, and quality of life was also reported. Brodalumab was generally well tolerated with no serious adverse effects. What is more, a significant decrease in vascularity and inflammation measured by cutaneous Doppler ultrasonography was seen compared with the baseline in each patient. In another open-label cohort study, ten patients were also injected with brodalumab 210 mg/1.5 mL subcutaneously; however, unlike in the above-mentioned research, it was administered weekly until week 24 [70]. A complete HiSCR response was observed at week 4.

Some promising results also come from a case report of a 45-year-old male presenting with recalcitrant gluteal hidradenitis suppurativa [71] The patient was administered brodalumab 210 mg subcutaneously at weeks 0, 1, and 2 followed by 210 mg for every 2 weeks. After 12 weeks of treatment, a significant reduction in the number of lesions, pain, discharge, and smell was observed. Furthermore, the signs of laboratory systemic inflammation also decreased. During 9 months of the patient’s follow-up, no relapse was noticed.

Presently, there is only one ongoing early phase I clinical trial trying to characterize the molecular response to brodalumab treatment and identify blood and tissue markers that reflect the severity of the disease [72].

#### 3.3.4. Ixekizumab

Ixekizumab (IXE) is a humanized IgG4 monoclonal antibody that neutralizes soluble IL-17A and IL-17 A/F. It has been approved by the FDA for the treatment of plaque psoriasis, psoriatic arthritis, ankylosing spondylitis, and non-radiographic axial spondylarthritis with objective signs of inflammation [73]. The recommended dose of the drug is 160 mg at week 0, followed by 80 mg every 2 or 4 weeks depending on the disease. The only data on the use of ixekizumab in HS come from case reports; nonetheless, the results suggest a considerable potential of the drug. In 2020, two case reports of acne inversa patients with concomitant psoriasis treated with IXE were published [74,75]. The typical dosing regimen was applied. Both of the reports suggest IXE as a safe and effective therapy for psoriasis and HS, however, indicating the need of further research in this area.

Another case report from 2021 described a female HS patient with concomitant herpes simplex virus (HSV) infection [76]. The patient was administered subcutaneous ixekizumab 160 mg at week 0, and 80 mg at weeks 2, 4, 6, 8, 10, and 12, followed by 80 mg every 4 weeks. Thirteen months after the treatment with IXE, the patient demonstrated a significant improvement in her disease. Thus, the authors postulated that IXE may come as another promising therapeutic option for patients with acne inversa. A summary of the significant clinical studies concerning the IL-17 inhibitors in HS are presented in Table 2.

### 3.4. IL-12/23 Inhibitors

#### Ustekinumab

Ustekinumab (UST) is a human IgG1κ monoclonal antibody against the p40 subunit of IL-12 and IL-23. What is more, according to a study by Adedokun et al., it has the ability to downregulate IL-8, IFN-γ, IFN-γ inducible protein-10, and monocyte chemoattractant protein (MCP)-1 [77]. It is FDA-approved for use in moderate to severe plaque psoriasis, active psoriatic arthritis, moderately to severely active Crohn’s disease, and moderately to severely active ulcerative colitis [78].

A phase II open-label study by Blok et al. enrolled 17 patients with moderate to severe HS [79]. The subjects were treated with either 45 or 90 mg of UST subcutaneously at weeks 0, 4, 16, and 28. As much as 82% of patients achieved a moderate-to-marked improvement of the modified Sartorius score at week 40 and the hidradenitis suppurativa clinical response (HiSCR) was met in 47% of subjects. Moreover, patients described as good responders to the biologic had a milder disease and a lower expression of leukotriene A4-hydrolase (LTA4H), which may indicate the effectiveness of UST.

In a multicentric retrospective review, the authors presented the results of the treatment of 14 HS patients with UST [80]. Patients were administered an intravenous infusion of the biologic adjusted by weight (≤55 kg, 260 mg; 55–85 kg, 390 mg; ≥85 kg, 520 mg), and then a subcutaneous dose of 90 mg every 8 weeks. Half of the treated patients achieved hidradenitis suppurativa clinical response (HiSCR) and 71.42% of them reached more than a 30% decrease in the dermatology life quality index (DLQI) and visual analog scale (VAS) of pain at week 16. Further investigation in randomized controlled trials is highly needed to determine the appropriate treatment dose for patients with HS.

### 3.5. IL-23 Inhibitors

#### 3.5.1. Guselkumab

Guselkumab is a human IgG1κ monoclonal antibody against the p19 subunit of IL-23. It has been approved by the FDA for use in moderate to severe plaque psoriasis in adults [81]. The recommended dose is 100 mg subcutaneously at week 0, week 4, and every 8 weeks thereafter and data obtained in case reports of HS patients support this regimen [82,83,84].

In a phase II placebo-controlled, double-blind study, 184 patients with moderate to severe HS were randomized to receive either 200 mg subcutaneously or 1200 mg intravenously of guselkumab, or a placebo [85]. At week 16, HiSCR was achieved in 50.8% of patients treated with 200 mg of guselkumab subcutaneously. From patients who received 1200 mg of guselkumab intravenously at weeks 0, 4, and 8, followed by 200 mg of guselkumab subcutaneously at week 12, 45% achieved HiSCR at week 16. In the placebo group, 38.7% of the participants met HiSCR.

#### 3.5.2. Risankizumab

Risankizumab is a fully IgG1κ monoclonal antibody that targets the IL-23. Case reports support the use of risankizumab in hidradenitis suppurativa [86,87,88]; however, larger clinical trials are required to prove its safety and efficacy in the disease.

There was only one phase 2, multicenter, randomized, placebo-controlled, double-blind trial to evaluate the safety and efficacy of risankizumab in hidradenitis suppurativa [89]. The study included two treatment periods. In period A, patients were randomized to receive either risankizumab dose A, dose B, or placebo, and in period B, subjects who received risankizumab dose A or placebo during period A received risankizumab dose B. Patients who received risankizumab dose B in period A remained on that dose in period B. However, no results have been published yet. A summary of significant clinical studies concerning the IL-17 inhibitors in HS are presented in Table 3.

### 3.6. Complement C5a Inhibitors

#### 3.6.1. Vilobelimab

Vilobelimab (IFX-1) is an IgG4κ monoclonal antibody binding to C5a and blocks its biological activity. The first open-label trial on the efficacy of IFX-1 in HS enrolled 12 patients [90]. The authors reported that after 50 days, 75% of patients treated with IFX-1 achieved HiSCR. Additionally, after 134 days, the number increased to 83.3%.

In the phase II controlled trial, the patients with moderate to severe HS were randomized to receive either 400 mg of IFX-1 intravenously every 4 weeks, 800 mg every 4 weeks, 800 mg every 2 weeks, or 1200 mg every 2 weeks, or a placebo [91]. The HiSCR response rate as high as 51.5% was met in the IFX-1 800 mg intravenously every 4 weeks group.

#### 3.6.2. Avacopan

Avacopan is a small molecule C5a receptor antagonist administered orally. A randomized, double-blind, placebo-controlled, parallel group, phase 2 study in patients with moderate to severe hidradenitis suppurativa investigated the safety and efficacy of avacopan in the disease [92]. The study included three randomized groups, 1:1:1, respectively, in the placebo, avacopan 10 mg twice daily, and avacopan 30 mg twice daily for 12 weeks. The results of the trial are yet to be published.

### 3.7. CD20 Inhibitor

#### Rituximab

Rituximab (RIX) is a chimeric mouse/human monoclonal antibody that targets the CD20 protein. It has been approved by the FDA for the treatment of non-Hodgkin’s lymphoma (NHL), chronic lymphocytic leukemia (CLL), rheumatoid arthritis (RA), granulomatosis with polyangitis (GPA), microscopic polyangitis (MPA), and moderate to severe pemphigus vulgaris [93]. The dose regimen varies from the disease.

In 2018, a case report of a successful treatment of HS with RIX in a patient with idiopathic carpotarsal osteolysis was published [94]. The patient also suffered from chronic active antibody-mediated rejection (CAAMR). RIX was administered in low doses of 200 mg intravenously in two courses. A dramatic improvement in HS was observed, without remission.

### 3.8. CD40 Inhibitor

#### Iscalimab (CFZ533)

Iscalimab (CFZ533) is a human monoclonal antibody that has the ability to block the CD40 pathway and the activation of CD40+ cell types. Currently, a phase II clinical study to evaluate the efficacy and safety of iscalimab in patients with moderate to severe HS is being conducted [95]. The trial is planned to be finished in mid-2023.

### 3.9. Phosphodiesterase-4 (PDE-4) Inhibitor

#### Apremilast

Apremilast is a selective phosphodiesterase-4 (PDE-4) inhibitor. It reduces the production of TNF, IL-12p40, and IL-17 by blocking the cyclic adenosine monophosphate (cAMP) degradation. Apremilast is approved by the FDA for the use in active psoriatic arthritis and moderate to severe plaque psoriasis [96].

In a case series study of nine HS patients who failed to respond to other treatments, subjects were administered oral apremilast 30 mg twice a day [97]. Five out of the six patients showed a significant improvement in the visual analog scale (VAS) in pain, Sartorius score clinical staging, and dermatology life quality index (DLQI) scores.

In one randomized clinical trial, twenty patients with mild to moderate HS were randomized in a 3:1 ratio [98]. Hence, 15 patients were administered 30 mg of apremilast twice daily and five patients received a placebo. In the apremilast group, 53.3% of patients achieved HiSCR after 16 weeks and none of the patients in the placebo group. What is more, a significant difference was observed in the abscess and nodule count (*p* = 0.011) in the study group.

After the study, participants who achieved HiSCR (*n* = 8) were offered to continue the treatment with apremilast [99]. Out of the eight initial patients, four discontinued the treatment within the first year. During the 2-year follow-up, all four remaining patients maintained HiSCR.

### 3.10. Anti-IL-36 Agents

#### 3.10.1. Spesolimab

Spesolimab is a monoclonal IgG1κ receptor antibody against IL-36 that upregulates neutrophil recruitment, the proliferation of keratinocytes, dendritic cells activation, and the secretion of pro-inflammatory mediators such as IL-1ß, TNF-α, IL-6, and IL-8 [100]. Although most of the data on spesolimab comes from the studies on pustular psoriasis, it is currently under investigation in hidradenitis suppurativa. In the phase II clinical trial, 52 patients were randomized to receive either a placebo or the treatment with intravenous spesolimab [101]. The results are yet to be published.

At the time of writing, there is one phase II clinical trial underway investigating the safety and efficacy of spesolimab in HS, which is a continuation of the above-mentioned research [102].

#### 3.10.2. Imsidolimab

Imsidolimab is a human monoclonal antibody that inhibits the activation of the IL-36 receptor. Currently, there is one phase II, randomized, double-blind, placebo-controlled clinical trial evaluating the efficacy and safety of imsidolimab in hidradenitis suppurativa [103].

### 3.11. Leukotriene A4 (LTA4) Inhibitor

#### LYS006

LYS 006 is a LTA4 hydrolase inhibitor administered orally. Presently, it is under investigation in a phase II clinical trial in patients with moderate to severe hidradenitis suppurativa. The study is supposed to be completed in mid-2023 [95].

### 3.12. Janus Kinase (JAK) Inhibitors

#### 3.12.1. INCB054707

INCB054707 is a Janus kinase (JAK) inhibitor that has already been studied in two multicentre phase II clinical trials, the results of which were published in early 2022 [104]. In study 1, 10 patients received open-label INCB054707 15 mg once daily. In study 2, the participants were randomized to 30, 60, or 90 mg of INCB054707, or placebo for 8 weeks. Safety and tolerability were the primary endpoints for both studies. Secondary endpoints included the hidradenitis suppurativa clinical response (HiSCR), the DLQI, the pain score, and the international hidradenitis suppurativa severity score system (IHS4). Out of the enrolled patients, 3 in study 1, and 17 receiving INCB054707 compared with four patients receiving placebo in study 2, achieved HiSCR at week 8. Moreover, the patients reported improvements in QoL, IHS4, and skin pain that were maintained over the treatment period.

Furthermore, it is being evaluated in ongoing clinical trials regarding its efficacy and safety in HS [105].

#### 3.12.2. Tofacitinib

Tofacitinib, a JAK inhibitor, is known to suppress inflammatory cytokines involved in the pathogenesis of acne inversa [106]. Its promising results have been already shown in a case report by Savage et al [107]. Two HS patients, unresponsive to previous therapies, were treated with 5 mg of tofacitinib twice daily in combination with other drugs often used in HS (cyclosporine (cyclosporine and amoxicillin for patient 1, and amoxicillin, amoxicillin-clavulanate, azithromycin, MMF, and triamcinolone ointment for patient 2)). No serious adverse reactions have been reported.

#### 3.12.3. Upadacitinib

Upadacitinib is another JAK inhibitor that was evaluated in a phase 2, randomized, placebo-controlled, double-blind trial in patients with moderate to severe hidradenitis suppurativa [108]. The participants were given doses of oral upadacitinib or placebo once daily for 48 weeks; nevertheless, the results have not been published yet.

### 3.13. CXC Receptors (CXCR1 and CXCR2)

#### LY3041658

LY3041658 is a monoclonal antibody that has the ability to neutralize chemokines binding to the CXCR1 or CXCR2 receptors. When activated, CXCR1 and CXCR2 mediate neutrophil recruitment and trigger cytotoxic effects at infection sites [109]. Since HS is considered a neutrophil-driven disease, this pathway seems promising.

Currently, there is an ongoing phase II randomized controlled study to evaluate the efficacy of intravenous LY3041658 in 52 patients with moderate to severe acne inversa [110]. A summary of immunomodulatory drugs and inhibited molecules, as well as ongoing clinical studies with biologics in HS are presented in Table 4 and Table 5.

## 4. Material and Methods

This study is a narrative review. The PubMed database has been searched for articles relevant to the presented review. The analysis reviewed the papers published by July 2022. Search terms included “hidradenitis suppurativa” or “acne inversa” and “immunomodulatory treatment” or “biological therapy” or “immunomodulation”. Duplicate articles, and those wrote in any other language than English, were excluded. The obtained articles were analyzed by content and quality analysis and compared. Additionally, a ClinicalTrials.gov site was searched for research studies regarding the immunomodulatory treatment of HS.

## 5. Conclusions

Nowadays, there are numerous treatment options available for patients with hidradenitis suppurativa. However, since the treatment often turns out to be ineffective, novel therapies are in high need to be evaluated. The safety and efficacy of adalimumab, still the only FDA-approved biologic in the HS treatment, paved the way for new candidates to be compared with adalimumab. Several more drugs with new immunological targets such as IL-12/23, IL-17, IL-23, IL-36, C5a, CD-20, CD-40, LTA4, and CXCR1/2 are presently under investigation for the treatment of acne inversa. Larger clinical trials on novel therapeutic agents to assess the efficacy and the safety of new drugs are mandatory in order to discover effective treatment options for HS patients and to hopefully improve their quality of life.

## Figures and Tables

**Table 1:** A summary of significant clinical studies concerning the IL-1 inhibitors in HS.

Authors	Biologic Drug	Dosage Regimen	Study Type	Efficacy
Tzanetakou, V. et al. [52]	Anakinra	100 mg s.c. for 12 weeks	Double-Blind, Randomized, Placebo-Controlled Clinical Trial	HiSCR was achieved in 78% of patients
Gottlieb, A. et al. [53]	Bermekimab	400 mg s.c. every week	Open Label Trial	After 12 weeks, HiSCR was achieved in 63% of patients in group A and 61% of patients in group B
Kanni T., et al. [54]	Bermekimab	7.5 mg/kg i.v. every 2 weeks	Phase II Randomized Clinical Trial	HiSCR was achieved in 60% of patients
Sun, N.Z., et al. [56] Jaeger T. et al. [57] Houriet C. [58]	Canakinumab	150 mg s.c. every week/4 weeks/8 weeks	Case Reports	Varied Results

**Table 2:** A summary of significant clinical studies concerning the IL-17 inhibitors in HS.

Authors	Biologic Drug	Dosage Regimen	Study Type	Efficacy
Prussick, L. et al. [60]	Secukinumab	Week 0, 1, 2, 3, 4—300 s.c. mg from week 8—300 mg s.c. every 4 weeks	Open Label Trial	At week 24, 78% of patients achieved HiSCR
Casseres, R.G. et al. [61]	Secukinumab	Week 0, 1, 2, 3, 4—300 mg s.c. from week 6/8—300 mg s.c. every 2/4 weeks	Open Label Trial	At week 24, 70% of patients achieved HiSCR
Glatt, S. et al. [65]	Bimekizumab	Week 0—640 mg s.c. from week 2—320 mg s.c. every 2 weeks	Double-Blind, Placebo-Controlled, Phase 2 Randomized Clinical Trial	At week 12, 57.3% of patients achieved HiSCR
Frew, J. W. et al. [69]	Brodalumab	Week 0, 1, 2—210 mg s.c. from week 4—210 mg s.c. every 2 weeks	Open Label Trial	100% of patients achieved HiSCR
Frew, J. W. et al. [70]	Brodalumab	210 mg s.c. every week	Open Label Trial	100% of patients achieved HiSCR
Arenbergerova, M. [71]	Brodalumab	Week 0, 1, 2—210 mg s.c. followed by 210 mg s.c. every 2 weeks	Case Report	After 12 weeks a significant reduction in number of lesions, pain, discharge, and smell was obtained
Odorici, G. et al. [74]	Ixekizumab	Week 0—160 mg s.c., week 2, 4, 6, 8, 10, 12—80 mg s.c. from week 16—80 mg s.c. every 4 weeks	Case Report	Satisfactory results
Megna, M. et al. [75]	Ixekizumab	Week 0—160 mg s.c., week 2, 4, 6, 8, 10, 12—80 mg s.c. from week 16—80 mg s.c. every 4 weeks	Case Report	Satisfactory results
Reardon, K. et al. [76]	Ixekizumab	Week 0—160 mg s.c., week 2, 4, 6, 8, 10, 12—80 mg s.c. from week 16—80 mg s.c. every 4 weeks	Case Report	A significant improvement in the disease

**Table 3:** A summary of significant clinical studies concerning the IL-23 inhibitors in HS.

Authors	Biologic Drug	Dosage Regimen	Study Type	Efficacy
Kovacs, M. et al. [82] Kearney, N. et al. [83] Jørgensen, A.-H.R. et al. [84]	Guselkumab	Week 0—100 mg s.c. from week 4—100 mg every 8 weeks	Case Reports	Varied Results
Janssen Research and Development [85]	Guselkumab	Week 0, 4, 8, 12—200 mg s.c. or week 0, 4, 8—1200 mg i.v., week 12—200 mg s.c.	Phase II Placebo-Controlled, Double-Blind study	At week 16, HiSCR was achieved in 50.8% of patients treated with 200 mg of guselkumab s.c. From patients who received 1200 mg of guselkumab i.v. followed by 200 mg of guselkumab s.c., 45% achieved HiSCR at week 16
Licata, G. et al. [86] Marques, E. et al. [87] Caposiena Caro, R.D. et al. [88]	Risankizumab	Week 0, 4—150 mg s.c. from week 16—150 mg every 12 weeks	Case Reports	Satisfactory Results

**Table 4:** A summary of immunomodulatory drugs and inhibited molecules.

TNF-α inhibitors	Adalimumab
	Infliximab
	Etanercept

	Golimumab
	Certolizumab
IL-1 inhibitors	Anakinra
	Bermekimab
	Canakinumab
IL-17 inhibitors	Secukinumab
	Bimekizumab
	BrodalumabIxekizumab
IL-12/23 inhibitors	Ustekinumab
IL-23 inhibitors	Guselkumab
	Risankizumab
Complement C5a inhibitors	Vilobelimab (IFX-1)Avacopan
CD20 inhibitor	Rituximab
CD40 inhibitor	Iscalimab (CFZ533)
Phosphodiesterase-4 (PDE-4) inhibitor	Apremilast
Anti-IL-36 agents	Spesolimab
	Imsidolimab
Leukotriene A4 (LTA4) inhibitor	LYS006
Janus Kinase (JAK) inhibitors	INCB054707
	Tofacitinib
	Upadacitinib
CXC receptors (CXCR1 and CXCR2)	LY 3041658

**Table 5:** A summary of ongoing clinical studies with biologics in HS.

Biologics	Clinical Trial Phase	The Estimated Date of Completion of the Trial	Estimated Size of the Study Group
CSL324	1	November 2022	40
Spesolimab	2	May 2024	45
Lutikizumab	2	December 2023	160
Imsidolimab	2	April 2023	120
Bermekimab	2	April 2024	290
Izokibep	2	February 2024	180
Bimekizumab	3	May 2023	505
